# The influence factors of medical disputes in Shanghai and implications - from the perspective of doctor, patient and disease

**DOI:** 10.1186/s12913-022-08490-5

**Published:** 2022-09-07

**Authors:** Yu Liu, Pei Wang, Yonghai Bai

**Affiliations:** 1 Department of Psychology, Education College, Shanghai Normal University, Shanghai, 200234 China; 2 Medical Department, Shanghai General Hospital, Shanghai Jiao Tong University School of Medicine, Shanghai, 200080 China; 3 School of Teacher Education, Honghe University, Mengzi, 661199 China; 4 Faculty of Education, East China Normal University, Shanghai, China; 5 Department of Medical Psychology, Second Affiliated Hospital of Naval Medical University, Shanghai, 200003 China

**Keywords:** Medical dispute, Dispute level, Factor of doctor, Factor of patient, Factor of disease

## Abstract

**Objective:**

This study aimed to explore the causes and factors behind medical disputes that occurred across eight hospitals in Shanghai over a three-year period (January 2018 to December 2020), thus providing targeted suggestions for amelioration.

**Methods:**

Stratified sampling was employed to collect 561 cases in which medical disputes occurred at two tertiary hospitals, two secondary hospitals, and four primary hospitals in Shanghai. The causes were analyzed using descriptive statistics, while the factors affecting the dispute level (i.e., 1 through 4, with 1 being most severe) were analyzed via one-way ANOVA and logistic regression analyses.

**Results:**

Doctors and patients variously contributed to the medical disputes; 86.1% were related to doctors, while 13.9% were related to patients. For doctors, there are seventeen factors that influenced medical disputes. In particular, the insufficient communication (28.82%) is the most prominent factor in the doctors’ factors. For patients, there are seven factors that influenced medical disputes. In particular, the misunderstanding of medical behavior (43.48%) is the most prominent factor in the patients’ factors. Of all investigated medical disputes, 406 were level 4 (78%), 95 were level 3 (18%), and 19 were level 2 (4%); there were no level 1 disputes. The reasons for different level placements included the disease classification, treatment effect, diagnosis and treatment regulation violations by doctors, and low technical levels.

**Conclusions:**

In addition to strengthening training about clinical and communication skills, the hospitals should establish quality control mechanisms for case records and construct rapid, standardized referral mechanisms. The doctors should attach great importance to the quality and urgency of treatment given to critically ill patients, who must be informed about their prognoses in a timely manner to avoid medical disputes and physical deterioration. The patients should actively cooperate with their doctors in the treatment process, moderate any unrealistic expectations that patients may have about the outcomes. During the COVID-19 pandemic particularly, doctors and patients should strengthen empathy and mutual trust more, then defeat disease together.

**Supplementary Information:**

The online version contains supplementary material available at 10.1186/s12913-022-08490-5.

## Introduction

Medical disputes usually refer to disputes between doctors and patients who have inconsistent understanding of the consequences of medical treatments and their causes, which maybe then brought to the health administrative department or judicial authority for accountability or compensation for losses. Medical disputes include civil disputes (civil compensation), administrative disputes (administrative punishment) and criminal liability (crime of medical malpractice) between doctors and patients in the broad sense. Based on whether there is a fault in the process of diagnosis and treatment, medical disputes can be divided into medical disputes with or without faults. Medical disputes with faults refer to those caused by medical malpractice and errors, and medical disputes without faults are caused by medical accidents and complications. Medical disputes also can be divided into iatrogenic disputes and non-iatrogenic disputes based on whether they are caused by iatrogenic reasons or not. The medical complaints are a common phenomenon in China. There are many medical complaints in the hospital every day and they are resolved through negotiations between doctors and patients or the administrative department in the hospital. When a complaint can not be resolved by negotiations between doctors and patients or the administrative department in the hospital, it will turn into a medical dispute. The medical dispute is a very sharp contradiction. There is a conceptual difference with a complaint that medical disputes are the products of contradictions intensified to a certain stage, and they will be brought to the health administrative department of region or judicial authority for accountability or compensation for losses. The doctor-patient relationship is at the core of interpersonal interaction in the medical setting, and is thus crucial for ensuring healthcare delivery. Specifically, the doctor-patient relationship consists of direct interactions between the care provider and care user [1]. However, the two parties have different understandings of the disputed facts, dispute with each other and express their opinions. The settlement of medical disputes is generally handled through consultation between doctors and patients. If the consultation fails, the parties may apply for mediation to the health administration department or directly file a civil lawsuit to the people’s court [2].

The doctor-patient relationship is generally harmonious in developed countries such as, where there are relatively comprehensive laws and medical insurance systems. However, the United Kingdom and United States experience high rates of dispute across medical institutions [3]. In 2005, Michigan Medicine conducted a workplace violence survey among emergency physicians, finding that 74.9% suffered verbal threats at least once per year, with 28.1% suffering physical violence within the year [4]. In developing countries, a survey showed that more than half of sampled medical staff had experienced medical disputes at least once [5]. In sum, the literature shows that doctor-patient disputes now constitute a global issue [6].

In China, the number of medical disputes has surged since the beginning of the twenty-first century [7]. Nationally, medical disputes have increased by an annual rate of 22.9% since China implemented the Medical Malpractice Management Regulation in September 2002 [8]; in some regions, this increase has reached 40% [9]. According to data released by the National Health Commission of China, there were 73 million outpatients in medical institutions across the nation as of 2015, with approximately 70,000 of these cases ultimately ending in medical disputes. In 2017, there were 12,734 medical disputes, followed by a slight decrease to 12,249 in 2018 and subsequent increase to 18,112 in 2019 [10]. As an international financial center, the city of Shanghai contains some of the richest medical resources in China, but the number of medical disputes is rising at an annual rate of 11% [11]. The collective dedication of health workers during COVID-19 has once again provided us a greater understanding of medical profession. At present, although the doctor-patient relationship in China is improving as a whole, there are still some disharmonious phenomena [12]. Domestic scholars tend to focus on the doctor-patient relationship in six main areas: medical science, hospital management research, health care ethics, public relations research, legal system and conflict resolution mechanism. On the other hand, foreign scholars tend to concentrate on the modes of doctor-patient relationships [13].

At present, the main influencing factors of medical disputes in China can be summarized as doctor’s factors, patient’s factors, social’s factors and so on. Among them, doctor’s factors mainly include diagnosis and treatment quality, insufficient communication, service attitude, awareness of responsibility, medical supplies and equipment, medical ethics and so on. The patient’s factors include misunderstanding of medical behavior, unreasonable appeal, high expectation of prognosis, refuse to cooperate with treatment, mistrust and so on. Social causes mainly include related laws and regulations are not matched, media reports on medical disputes are not objective and fair, the total amount of medical resources is insufficient, the regional distribution is uneven, the mechanism of medical dispute settlement is not perfect and so on [14].

The scholars in the developed countries such as Britain and Amercia researched medical disputes earlier, they researched the doctor-patient relations firstly [15]. Beckman HB once pointed out that 70% of medical disputes came from insufficient communication, the sufficient communication between doctor and patient is a strong guarantee to prevent medical disputes, and the insufficient communication is the main factor affecting medical disputes [16]. Japan is a country with a relatively low incidence of medical disputes in the world. The Japanese scholars thought that hospital management, respect for informed consent of patients, the establishment of an effective medical liability insurance system, and the establishment of the perfect system of medical dispute settlement were important reasons that affected medical disputes [17]. Some scholars also put forward that cultural background, educational background and other humanistic factors were important factors that led to medical disputes. The mainstream scholars thought the sufficient communication between doctor and patient can help make up for the two sides of the differences in understanding, and then prevented the formation of medical disputes [14].

Now, there are many researches on doctor-patient relationship, most of which are qualitative researches. The quantitative researches are few from the perspective of hospital management with COVID-19.This study aimed to explore the causes and factors behind medical disputes that occurred across eight hospitals in Shanghai over a three-year period (January 2018 to December 2020), analyze the risk factors, thus providing targeted suggestions for amelioration. We believe that our findings will both provide a resource that hospitals in Shanghai can use to prevent medical disputes and serve as a foundation for the further research on doctor-patient relationship.

## Methods

This study innovatively took hospital management as the cut-in point; based on the perspectives of doctors and patients as well as disease-related factors, we analyzed information from 561 medical disputes that occurred in Shanghai over a three-year period (January 2018 to December 2020), as extracted via multistage sampling. Specifically, we analyzed the high-risk factors for disputes and conducted a correlation test to determine which influencing factors were likely to further escalate disputes.

### Study sample

We initially employed multistage sampling to collect information on 561 medical disputes that occurred in two Class A tertiary hospitals, two Class A secondary hospitals, and four community hospitals in Shanghai over a three-year period (2018 to 2020). The Class A tertiary hospital aims to diagnosis and treat the difficult and critical diseases. The Class A secondary hospital aims to diagnosis and treat the common diseases. The community hospital aims to treat and manage the chronic diseases. Of these, 41 cases were removed due to incomplete information, resulting in 520 cases with complete information for analysis (pass rate of 92.69%).

### Research measures

As previously developed by the current research team, this study used the Questionnaire on Medical Dispute Case Analysis [13], which is comprised of six dimensions covering 23 items, including demographic indicators (six items), medical factors (four items), patient factors (two items), disease factors (four items), communication factors (two items), and dispute handling factors (five items).

The demographic indicators include gender, age, native place, occupation, education, marriage. The medical factors include attending doctor, medical quality, expert opinion, non-technical factor. The expert opinions of medical factors include violation of diagnosis and treatment regulation, belated diagnosis and treatment, imperfect operation, low technical level and so on. The patient factors include medical insurance and non-error medical disputes factors. The non-error medical disputes factors of patient factors include misunderstanding of medical behavior, bad attitude, mistrust, inadequate medical knowledge and so on. The communication factors include doctor’s factors and patient’s factors. The doctor’s factors of communication factors include critical behavior to patients, insufficient communication and others. The patient’s factors of communication factors include bad attitude, patient’s speech threatened the door and so on. The dispute handling factors include dispute level, amount of compensation, handling time, violent conflict and so on. The dispute level include Level1, Level2, Level3, Level4.The amount of compensation include above one million REN MIN BI, between 500,000 and one million REN MIN BI, between 100,000 and 500,000 REN MIN BI, below 100,000 REN MIN BI. This classification method comes from the research that was conducted by Yonghai Bai about influencing factors of medical disputes in Class A tertiary hospital in Shanghai [13].

### Procedures

We conducted a retrospective analysis and processed documents related to the obtained cases. Prior to the investigation, we requested that the hospital president in charge of medical disputes help communicate with the director of reception office and we trained the investigators. The researcher introduced the plan, the purpose, the method of the research, the situation related to the questionnaire to all the investigators, so that the investigators can have an overall understanding of the project, and we explains the steps, requirements, time arrangement, workload and other specific issues of the questionnaire. We trained the investigators to make them clear about all the contents of the questionnaire, the way of filling and the items need to be concerned of the questionnaire. According to the requirements and steps of the formal investigation, we conduct a simulation investigation and let each investigator practice from beginning to end. Then, we summarized the problems in the simulation investigation and solved these problems through discussion or explanation. The discussion or explanation include that organizational management measures, guidance and supervision measures, review and inspection measures, summary and exchange system.

Then we went to the hospital medical office of the hospital and the director of the dispute office helped find the case files of medical disputes from January 2018 to December 2020. The investigators first read and analyzed the medical dispute cases, and then according to the questions in the questionnaire, he extracted information about doctors, patients and diseases from the medical cases. At the same time, he filled in the questionnaire timely. In the questionnaire, there is an option for the amount of compensation for medical disputes. In the medical disputes files, there is the amount of compensation for disputes. We extracted the amount of compensation information from the case of medical disputes and filled in the questionnaire. Once one questionnaire was filled out, another investigator performed strict double-check task. During this process, all questions that we asked were answered timely by the director of the dispute office. After the investigation in one hospital, we carried out the same investigation in another hospital, and a total of eight hospitals were investigated.

### Data analysis

The data collected thereby collected were recorded via Microsoft Excel and processed using IBM SPSS18. Diseases were divided into four categories in line with the principle of case classification [18]; this included simple general cases, simple emergent cases, complex intractable cases, and complex critical cases. Medical disputes were classified into one of four levels, including level 4 (compensation below 100,000 REN MIN BI), level 3 (compensation between 100,000 and 500,000 REN MIN BI), level 2 (compensation between 500,000 and one million REN MIN BI), and level 1 (compensation above one million REN MIN BI) [19]. Taking the disease-related, doctor-related, and patient-related factors as independent variables and the dispute levels as dependent variables, we conducted a one-way ANOVA to more thoroughly analyze the medical dispute levels. The results were then substituted into a multiple logistic regression model to obtain the indicators of high-risk factors for medical disputes (significance at 0.05).

## Results

### Factors that influence medical disputes

#### Factors related to doctors

For doctors, there are seventeen factors that influenced medical disputes (see Table 1 for details). Insufficient communication is the most main reason in medical disputes. Insufficient communication can easily lead to medical disputes. For Example, due to more intraoperative blood loss, a rectal cancer operation patient needs blood transfusion treatment. Because before the blood transfusion treatment, the doctor did not communicate with the patient about the potential risk of blood transfusion fully, so that when the serious reaction in the blood transfusion, the patient refused to accept the fact then it caused medical dispute.

**Table 1:** Doctor’s factors in medical disputes

Reason	Number of dispute (case)	Proportion (%)
Insufficient communication	179	28.82
Low technical level	105	16.91
Lack of sense of responsibility	55	8.86
Defective case records	43	6.92
Imperfect operation	40	6.44
Inadequate experience	40	6.44
Inadequate condition evaluation	36	5.80
Irregular management process	25	4.03
Violation of diagnosis and treatment regulation	25	4.03
Misdiagnosis and mistreatment	22	3.54
Belated diagnosis and treatment	16	2.58
Postoperative complications	15	2.42
Equipment problems	7	1.13
Missed diagnosis	5	0.81
Poor condition monitoring	3	0.48
Unreasonable charge	3	0.48
Poor service attitude	2	0.32

As shown in Table 1, the main reasons related to doctors in medical disputes were insufficient communication (28.82%), low technical level (16.91%). Here, insufficient communication and low technical level affected 45.73% of all cases, thus showing the need to prioritize these factors when attempting to ameliorate doctor-patient disputes.

#### Factors related to patients

For patients, there are seven factors that influenced medical disputes (see Table 2 for details). The misunderstanding of medical behavior is the most main reason. For example, when the maternal hemorrhoids bleed in the process of delivery, the doctor needs suture bleeding points urgently. The videos or photos of hemostatic treatment will be mistaken for “sewing the anal”. When the perineal tears during labor, the doctor needs suture the wound. This operation will be mistaken for “sewing the vagina”. These operations cause the patient^’^s misunderstanding easily. They can become the “criminal evidence” of doctors.

**Table 2:** Patient’s factors in medical disputes

Reason	Number of dispute (case)	Proportion (%)
Misunderstanding of medical Behavior	40	43.48%
High expectation of prognosis	23	25.00%
Bad attitude	12	13.04%
Inadequate medical knowledge	7	7.61%
Disturbance	6	6.52%
Poor compliance	3	3.26%
Mistrust	1	1.09%

Looking at Table 2, the misunderstanding of medical behavior and high expectations for prognosis affected 68.48% of all cases, and were thus the main causes for medical disputes stemming from patients.

#### Factors related to the disease classification

Here, the disease classifications and treatment effects were the main factors (see Table 3). The proportion of the simple general disease caused medical disputes is the most in disease classification. The proportion of death caused medical disputes is the most in treatment effect.

**Table 3:** Distribution of disease factors in medical disputes

**Disease classification**	Number of case	Proportion (%)
Simple general	300	57.69
Simple emergent	47	9.04
Complex intractable	115	22.12
Complex critical	58	11.15
**Treatment effect**		
Clinically cured	69	20.25
Remission	148	21.52
Aggravation	152	24.68
Death	151	33.54

### Analyzing the factors that influence medical dispute levels

Of the 520 total analyzed medical dispute cases, 406 were level 4 (78%), 95 were level 3 (18%), and 19 were level 2 (4%); none were level 1. Here, the vast majority were at the lower end of the compensation brackets, thus indicating more controllable circumstances.

#### The influence of medical disputes

Disease-related factors included the disease classifications and treatment effects, while the doctor-related factors included professional title, working years, qualifications, violations of diagnosis and treatment regulations, misdiagnosis and mistreatment, belated diagnosis and treatment, imperfect operation, insufficient condition evaluation, low technical level, inadequate experience, and defective case records, and the patient-related factors included the patient’s gender, age, native place, misunderstanding of medical behavior, high expectation for prognosis, bad attitude, inadequate medical knowledge, poor compliance, mistrust, and disturbance. Here, a one-way ANOVA showed statistical significance for the patient’s native place (χ^2^ = 12.60, *P* = 0.002), disease classification (χ^2^ = 55.861, *P* = 0.000), treatment effect (χ^2^ = 80.744, *P* = 0.000), doctor’s professional title (χ^2^ = 9.061, *P* = 0.011), violation of diagnosis and treatment regulation (χ^2^ = 66.956, *P* = 0.000), belated diagnosis and treatment (χ^2^ = 16.123, *P* = 0.000), insufficient condition evaluation (χ^2^ = 19.101, *P* = 0.000), inadequate experience (χ^2^ = 50.838, *P* = 0.000), low technical level (χ^2^ = 14.916, *P* = 0.001), and defective case records (χ^2^ = 12.950, *P* = 0.002).

#### The reasons for different medical dispute levels

The above results of the one-way ANOVA about the influence of medical disputes were then substituted into multiple logistic regression equations (significance at 0.05). Table 4 shows the detailed results.

**Table 4:** Multiple Logistic regression analysis of factors influencing medical dispute level

	B	SE	Wald	df	Sig
Level-2 medical dispute	-.831	.944	.776	1	.378
Level-3 medical dispute	1.595	.946	2.840	1	.092
Disease classification (simple general)	.915	.374	6.000	1	.014
Disease classification (simple emergent)	.342	.500	.468	1	.494
Disease classification(complex intractable)	-.155	.365	.182	1	.670
Disease classification (complex critical)	0^a^			0	
Treatment effect (cured)	2.252	.645	12.178	1	.000
Treatment effect (remission)	1.804	.391	21.268	1	.000
Treatment effect (aggravation)	.805	.299	7.227	1	.007
Treatment effect (death)	0^a^			0	
Violation of diagnosis and treatment regulation	1.174	.452	6.752	1	.009
Not violating diagnosis and treatment regulation	0^a^			0	
Low technical level	.674	.279	5.861	1	.015
High technical level	0^a^			0	

*Note.* Link function: Logit

^a^Parameter is set as zero as it is redundant

As shown, the disease classification, treatment effect, doctor’s violation of diagnosis and treatment regulations, and low technical level were the reasons for different medical dispute levels.

## Discussion

### The influence of doctors and implications

#### Enhancing professional training and improving clinical skills

Of all investigated medical disputes, 36% were caused by deficient clinical practices among doctors (e.g., misdiagnosis and mistreatment, belated diagnosis and treatment, imperfect operation, inadequate condition evaluation, low technical level, and insufficient experience). This is often because young doctors lack standardized professional training and are largely deficient in clinical practice, which diminishes service quality [20]. To reduce the potential for medical disputes, this highlights the need for hospitals to improve their regulations and rules for clinical diagnosis and treatment, actively launch standardized clinical pathways, and offer professional skills training for young doctors to advance their efficacy in clinical practice.

#### Improving communication skills and creating a harmonious environment

Next, we found that 26.25% of all considered medical disputes were caused by insufficient communication. Effective communication is the cornerstone of medical service, it can improve satisfaction and compliance among patients while enhancing treatment effect. Research has shown that the cultivation of effective communication skills is an important part of becoming a good doctor [21]. Hospital administrators should work to formulate various methods of enhancement communication at different stages of diagnosis and treatment and ensure that appropriate and effective training opportunities are offered to all staff. To bridge the gap between doctors and patients resulting from the professional nature of medicine, it is crucial to improve the communication skills of doctors [22]. In Order to prevent the transmission of the Novel Coronavirus pandemic, mild COVID-19 patients in China have been quarantined in mobile cabin hospital for treatment. There are various forms of communication in the cabin, group doctor-patient communication is an innovative form of daily medical activities. In this condition, patients are prone to anxiety, pessimism, panic, depression and other negative emotions. Doctor-patient communication is extremely important, and doctors should pay more attention to the cultivation of doctor-patient communication skills in future diagnosis and treatment [23].

#### Establishing a quality control mechanism for case records and improving record writing

There were also medical records factors, as 6.3% of all investigated medical disputes were affected by the quality of case records. This emphasizes the need for quality control, particular through a feedforward control measure in medical quality management. Hospital administrative departments should formulate strict case record quality control systems, implement a three-level quality monitoring method for handling case records, reinforce supervision and management practices for medical treatment coversheets, enforce terminal case record reviews, conduct random checks on case records from departments that are prone to medical disputes, and help young doctors focus on writing case records in a way that elevates medical service quality [24]. These measures should functionally work to reduce the occurrence of medical disputes.

### The influence of patients and implications

From the perspective of issues caused by patients, 68.48% of the investigated medical disputes were caused by the misunderstanding of medical behavior and high expectations for prognosis. In this regard, trust is both the cornerstone of the doctor-patient relationship and a measure of harmony. Research has shown that patients trust their doctors generally have more satisfactory treatments [25]. To protect their own rights and interests, patients whose health awareness have increased may frequently question medical behaviors at all stages of the medical process [26]. The mistrust between doctors and patients inevitably leads to misunderstandings about reasonable medical behaviors. As medical technology continues to develop at a rate that matches societal progress, patients also tend to have greater expectations for prescribed treatments effects. Those who do not understand the limitations of these treatments may be dissatisfied with the outcomes [27], thus increasing the potential for medical disputes. Although patients tend to prevail in these disputes, they are playing a negative sum game that will ultimately harm their own interests, because disease is the common enemy of doctors and patients. The distrust between doctors and patients will eventually affect doctors’ treatment of patients. For some urgent operations, doctors will consider from the perspective of maximizing their own interests to protect their own interests from encroachment by patients.

The patients should recognize the current limitations of medicine and understand that disease is the common enemy of all involved. In particular, under the current situation of COVID-19, patients should fully realize that doctors and patients are community of common future. It is inevitable that patients under the current situation of COVID-19 will have poor medical experience, suffer from anxiety and great mental stress during the epidemic. Under such circumstances, empathy between doctors and patients should be strengthened and enhance their trust [28]. The patients should approach the medical setting with trust in their doctors, and actively discuss treatment decisions with the goal of jointly fighting disease [29]. From the other end, doctors should fully explain treatment plans and work to communicate with their patients, thus addressing any misunderstanding about reasonable medical behavior. They should also emphasize the possible risks of treatment, which will help patients develop reasonable expectations about the outcomes.

### The influence of disease management and implications

The level of a given medical dispute was affected by both the disease classification and treatment effects. Curing disease and saving lives are the primary tasks of the hospital, while restored health is the ultimate expectation of the patient [11]. When a treatment effect is not ideal and the patient does not understand the limitations of the applied medical technology, they are highly likely to oppose the course of action prescribed by the doctor, thus leading to medical disputes or even direct dispute. Here, doctors should attach great importance to the quality of care given to critically ill patients. They should treat all such patients in strict accordance with clinical pathways, work to improve the treatment effects, and provide timely information about any prognoses. To better address the needs of some critically ill patients, hospitals should establish rapid and standardized referral mechanisms and arrange all referrals with the goal of protecting life and health.

### The implications of legal and insurance systems

A sound legal mechanism can effectively decrease ineffectiveness in doctor-patient communication, fundamentally reduce misunderstandings between doctors and patients, and protect the legal rights of doctors and patients. To improve Chinese legal mechanism and facilitate agreeable relationships between doctors and patients, it is important to improve the relevant resolution mechanisms and measures as well as improve the administrative coordination mechanism among other actions.

In terms of medical insurance, medical insurance institutions should strengthen the information guidance for insured persons, reduce the contradiction between doctors and patients caused by information asymmetry. The government should improve the medical insurance system, implement medical insurance bills to pay for diseases, increase the protection of difficult diseases and expand health insurance coverage.

## Conclusion

This study’s investigation of 520 medical disputes in Shanghai revealed that issues were variously influenced by doctors, patients, and disease. For doctors, the main factors included professional title, violations of diagnosis and treatment regulations, misdiagnosis and mistreatment, belated diagnosis and treatment, imperfect operations, insufficient condition evaluations, low technical levels, the lack of experience, and defective case records, among others. For patients, prominent factors included the misunderstanding of medical behavior and high expectations for prognosis. Finally, factors related to disease included the disease classification and treatment effects. Among all factors, the disease classification, treatment effects, doctor’s violation of diagnosis and treatment regulations, and doctor’s low technical levels were the main reasons for different dispute levels, and are thus high-risk factors that require close attention. In addition to strengthening clinical and communication skills training, hospitals should establish quality control mechanisms for case records and construct rapid, standardized referral mechanisms. In the interpersonal context, patients should actively cooperate with their doctors in the treatment process, moderate any unrealistic expectations that patients may have about the outcomes. Doctors should also attach great importance to the quality and urgency of treatment given to critically ill patients, who must be informed about their prognoses in a timely manner to avoid medical disputes and physical deterioration. During the COVID-19 pandemic particularly, doctors and patients should strengthen empathy and trust more, then defeat disease together.

## Limitations

Due to limited time and resources, this study focused on conditions in Shanghai. Specifically, we solely investigated disputes from eight medical institutions via multistage sampling. As such, the results cannot be generalized to all of Shanghai or in other locations. Future research should conduct a larger survey in Shanghai to more thoroughly describe the causes of medical disputes and verify which high-risk factors are most influential. Nevertheless, the current findings constitute important clinical guidance and should serve as a basis for continued research.  

## Supplementary Information

**Additional file 1.** The conflict of doctor-patient questionnaire.

## Data Availability

The data used and/or analysed during the current study available from the corresponding author on reasonable request.

## References

[1] Harbishettar V, Krishna K, Srinivasa P, Gowda M (2019). The enigma of doctor-patient relationship. Indian J Psychiatry.

[2] Yu LS (2015). Analysis of Forensic Autopsy in 120 Cases of Medical Disputes Among Different Levels of Institutional Settings. J Forensic Sci.

[3] Xing K (2016). Concern about workplace violence and its risk factors in chinese township hospitals: A cross-sectional study. Int J Environ Res Public Health.

[4] Acik Y (2008). Experience of workplace violence during medical speciality training in Turkey. Occup Med (Chic Ill).

[5] Zaremba LS, Smoleński WH (2000). Optimal portfolio choice under a liability constraint. Ann Oper Res.

[6] Morken T, Johansen IH, Alsaker K (2015). Dealing with workplace violence in emergency primary health care: A focus group study. BMC Fam Pract.

[7] Zeng Y, Zhang L, Yao G, Fang Y (2018). Analysis of current situation and influencing factor of medical disputes among different levels of medical institutions based on the game theory in Xiamen of China A cross-sectional survey. Med (United States).

[8] Yu F, Xie X, Ding F, Xue C, Sun Z (2018). Changing procedures for resolving medical disputes in China. Intern Med J.

[9] Yuan JJ (2016). Empirical study on methods for handling medical disputes in public hospitals. J Shanghai Jiaotong Univ Medical Sci.

[10] Zhang J (2020). Current situation and prevention measures of doctor-patient relationship in China under the new era. Labor Secur World.

[11] Yin L, Zeng RH, Gao X, G. J. D. Analysis on the status-quo and influencing factors of medical disputes in the III A Hospitals--based on the perspective of doctors, patients and family members. Health Econ Res. 2019,36:67–74.

[12] Zhao J, Sun M, Zou D (2022). Rethinking the doctor-patient relationship in COVID-19 and post-COVID-19 era. Chinese Hospital Mgt.

[13] Liu Y, Bai Y, Wang P, Xu Z (2018). Study of the factors causing medical disputes in a Third-Level Grade A Hospital in Shanghai. Int J Health Plann Manage.

[14] Bian C (2021). Analysis of influencing factors of medical disputes in a Third Class Hospital in Jilin Province [D].

[15] Ma J, Nie B (2012). The exploration of the training about doctor-patient communication ability of pediatric stomatology students [J]. Med Educ Res Pract.

[16] Beckman HB, Markakis KM, Suchman AL (1994). The Doctor-Patient Relationship and Malpractice. Arch Intern Med.

[17] Wang KeSi, Wan L (2009). Japanese medical dispute prevention and treatment measures [J]. Med Sci Phil.

[18] G, Y. Design of the Case Classification System Based on XGBoost Algorithm. China Digital Medicine. 2018;(3):69-71.

[19] Liu Y, Bai Y (2019). The influence factors of medical disputes in a tertiary hospital in Shanghai. Chinese Heal Qual Manag.

[20] Zhao S, Huang H, Wu M (2016). Analysis of influence factors of medical disputes in a tertiary hospital from 2009 to 2014. China Med Her.

[21] Sun C (2020). New doctor-patient communication learning software to help interns succeed in communication skills. BMC Med Educ.

[22] Korzh O, Tsodikova O (2019). Improving doctor-patient communication in a primary care setting. Rom J Med Pract.

[23] Tang S, Shao J, Wang J (2020). The role and enlightenment of doctor-patient communication in the diagnosis and treatment of patients with mild COVID-19. Med Ethcis in China.

[24] Zhu WJ, Yu SY, Wang D (2019). Discussion on cultivating medical staff’s doctor-patient communicative ability under the new situation. Soft Sci Heal.

[25] Gu L (2019). The impact of contract service policy and doctor communication skills on rural patient-doctor trust relationship in the village clinics of three counties. BMC Health Serv Res.

[26] Zhang Z, Xiong J, Wu S (2019). The violation and repair of doctor-patient trust from the perspective of medical—Exploratory analysis based on grounded theory. Syst Eng Theory Pract.

[27] Yan Y, Wang LQ, Si ZY, Wu YY, Ma D (2019). The analysis of the causes for poor doctor-patient communication and its implication for medical education. Med Philos.

[28] Yao B, Fu H, Chen Q (2021). Thoughts on building a doctor-patient community with a shared future in COVID-19. Chinese Med Ethics.

[29] Wei D, Xu A, Wu X (2020). The mediating effect of trust on the relationship between doctor–patient communication and patients’ risk perception during treatment. PsyCh J.

