# Supplementary material for: The influence factors of medical disputes in Shanghai and implications - from the perspective of doctor, patient and disease
Source: BMC Health Serv Res. 2022 Sep 7;22:1128. doi: 10.1186/s12913-022-08490-5 (PMC9449943; doi:10.1186/s12913-022-08490-5)
Supplement: Supplementary file 1 — Additional file 1. The conflict of doctor-patient questionnaire. [file 12913_2022_8490_MOESM1_ESM.docx]

The conflict of doctor-patient questionnaire

| Department Doctor Name | |  | | | |
| --- | --- | --- | --- | --- | --- |
| Patient name | Patient phone | | Patients address | | |
| Risk factors | | Yes(√) | | | |
| **1、 Demographic indicator** | |  | | | |
| Gender | | male | | | |
|  |  | female | | | |
| Age | | <20 | | | |
|  |  | 20-40 | | | |
|  |  | 40-60 | | | |
|  |  | >60 | | | |
| Native place | |  | | | |
| Occupation | | teacher | | | |
|  |  | company employee | | | |
|  |  | worker | | | |
|  |  | civil servant  medical staff | | | |
|  |  | peasant | | | |
|  |  | retiree | | | |
|  |  | soldier  others | | | |
|  |  |  | | | |
| Education | | bachelor degree or above | | | |
|  |  | technical secondary school or above | | | |
|  |  | junior high school graduates and above | | | |
|  |  | primary school and below | | | |
| Marriage | | Yes | | | |
|  |  | No | | | |
| **2、 Disease factors** | |  | | | |
| Congenerous disease | |  | | | |
| Attendance frequency | | once | | | |
|  |  | twice | | | |
|  |  | **≥**three times | | | |
| Classification of Diseases | | simple general | | | |
|  |  | simple emergent | | | |
|  |  | complex intractable | | | |
|  |  | complex critical | | | |
| Surgical classification | | level1 | | | |
|  |  | level2 | | | |
|  |  | level3 | | | |
|  |  | level4 | | | |
| Treatment effect | | clinically cured  remission | | | |
|  |  | aggravation | | | |
|  |  | death | | | |
| 3、 Doctor factors | |  | | | |
|  | | profession | | | chief Physician |
|  | |  |  |  | sssociate chief physician |
|  | |  |  |  | visiting staff |
|  | |  |  |  | resident doctor |
| Attending doctor | | working years | | | <10 |
|  |  |  |  |  | 10-20 |
|  |  |  |  |  | 20-30 |
|  |  |  |  |  | >30 |
|  |  | qualification or not | | Yes | |
|  |  |  |  | No | |
|  |  | length of stay | | <10  10-20  20-30 | |
|  |  |  |  | >30 | |
| Medical quality | | medical expense | | | |
|  |  | drug proportion | | | |
|  |  | violation of diagnosis and treatment regulation | | | |
|  |  | misdiagnosis and mistreatment | | | |
| Expert opinion | | belated diagnosis and treatment | | | |
|  |  | imperfect operation | | | |
|  |  | doctor is too optimistic about condition | | | |
|  |  | others | | | |
| Non-technical factor | | defective case records | | | |
|  |  | unreasonable charge | | | |
|  |  | lack of sense of responsibility | | | |
|  |  | not pay attention to patient privacy  others | | | |
| **4、Patient factors** | | | | | |
| Medical insurance | | Urban medical insurance | | | |
|  | | New Cooperative Medical System | | | |
|  | | Commercial insurance | | | |
|  | | No medical insurance | | | |
| Non-error medical disputes factors | | poor compliance | | | |
|  |  | misunderstanding of medical behavior | | | |
|  |  | high expectation to prognosis  bad attitude  mistrust  inadequate medical knowledge  others | | | |
| **5、 Communication factors** | |  | | | |
| Doctor factors | | critical behavior to patients/family members | | | |
|  | | insufficient communication  others | | | |
| Patient factors | | bad attitude | | | |
|  | | patient's speech threatened the doctor  others | | | |
| **6、 Dispute processing** | |  | | | |
| Dispute level | | Level1 | | | |
|  |  | Level2 | | | |
|  |  | Level3 | | | |
|  |  | Level4 | | | |
| Amount of compensation | | above one million REN MIN BI | | | |
|  | | between 500,000 and one million REN MIN BI | | | |
|  | | between 100,000 and 500,000 REN MIN BI | | | |
|  | | below 100,000 REN MIN BI | | | |
| Handling time (Day) | | <7 | | | |
|  |  | 7-30 | | | |
|  |  | 30-60 | | | |
|  |  | >60 | | | |
| violent conflict | | Yes | | | |
|  |  | No | | | |
| family member involvement | | High | | | |
|  | | Medium | | | |
|  | | Low | | | |
